# Substantia nigra dopaminergic neurons and striatal interneurons are engaged in three parallel but interdependent postnatal neurotrophic circuits

**DOI:** 10.1111/acel.12821

**Published:** 2018-07-30

**Authors:** Clara Ortega‐de San Luis, Manuel A. Sanchez‐Garcia, Jose Luis Nieto‐Gonzalez, Pablo García‐Junco‐Clemente, Adoracion Montero‐Sanchez, Rafael Fernandez‐Chacon, Alberto Pascual

**Affiliations:** ^1^ Instituto de Biomedicina de Sevilla, Hospital Universitario Virgen del Rocío/CSIC/ Universidad de Sevilla Seville Spain; ^2^ Departamento de Fisiología Médica y Biofísica Universidad de Sevilla, and CIBERNED Seville Spain; ^3^ Present address: School of Biochemistry and Immunology, Trinity Biomedical Sciences Institute Trinity College Dublin Dublin 2 Ireland

**Keywords:** GDNF, neurotrophic support, Parkinson’s disease, Shh, striatal interneurons, substantia nigra

## Abstract

The striatum integrates motor behavior using a well‐defined microcircuit whose individual components are independently affected in several neurological diseases. The glial cell line‐derived neurotrophic factor (GDNF), synthesized by striatal interneurons, and Sonic hedgehog (Shh), produced by the dopaminergic neurons of the substantia nigra (DA SNpc), are both involved in the nigrostriatal maintenance but the reciprocal neurotrophic relationships among these neurons are only partially understood. To define the postnatal neurotrophic connections among fast‐spiking GABAergic interneurons (FS), cholinergic interneurons (ACh), and DA SNpc, we used a genetically induced mouse model of postnatal DA SNpc neurodegeneration and separately eliminated *Smoothened* (*Smo*), the obligatory transducer of Shh signaling, in striatal interneurons. We show that FS postnatal survival relies on DA SNpc and is independent of Shh signaling. On the contrary, Shh signaling but not dopaminergic striatal innervation is required to maintain ACh in the postnatal striatum. ACh are required for DA SNpc survival in a GDNF‐independent manner. These data demonstrate the existence of three parallel but interdependent neurotrophic relationships between SN and striatal interneurons, partially defined by Shh and GDNF. The definition of these new neurotrophic interactions opens the search for new molecules involved in the striatal modulatory circuit maintenance with potential therapeutic value.

## 1 INTRODUCTION

Striatal circuits integrate both motor and cognitive cortical signals with motivational, salient, and reward‐related information from dopaminergic neurons of the substantia nigra (DA SNpc; Bolam et al., 2006). The central components of the striatal circuit are cortical glutamatergic terminals that activate striatal projection neurons (medium spiny neurons, MSN). Glutamatergic signaling is modulated by dopamine from DA SNpc cells in concert with the activity of local cholinergic interneurons (ACh) and fast‐spiking GABAergic interneurons (FS; Threlfell et al., 2010; Bonsi et al., 2011; Surmeier, Carrillo‐Reid, & Bargas, 2011; Silberberg & Bolam, 2015) that together constitute the striatal modulatory circuit. The relevance of these neuronal populations on striatal function is highlighted by the alteration of distinct neuronal types in several neurological disorders, including chorea (MSN), parkinsonism (DA SNpc), Tourette syndrome (ACh and FS), and dystonia (ACh). Although it is well known that acute alteration of a specific striatal component induces changes in the other elements of the circuit (Girasole & Nelson, 2015; Salin et al., 2009), knowledge on how chronic loss of a particular cell type disturbs the whole circuit's homeostasis is scarce.

**Figure 1:**
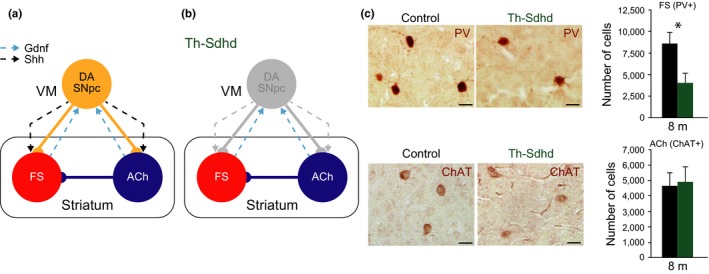
Striatal fast‐spiking (FS) but not cholinergic (ACh) interneurons require DA SNpc for postnatal survival. (a) Schematic representation of the striatal modulatory circuit. Solid lines represent neuronal connectivity and dashed lines neurotrophism. VM, ventral mesencephalon. (b) Schematic representation of the Th‐Shdh mouse model, where a degeneration of the DA SNpc cell bodies and projections is observed (Diaz‐Castro et al., 2012). (c) Brain coronal striatal sections immunostained with anti‐PV (upper panels), a marker of FS interneurons and anti‐ChAT (lower panels), a marker of ACh interneurons antibodies from 8‐month‐old control (left) and Th‐Sdhd (right) mice. Scale bars: 25 µm. Graphs represent the total striatal number of FS (upper panels) and ACh (lower panels) interneurons measured by stereological methods from control (black bars) and Th‐Sdhd (green bars) mice. In the entire figure, values represent mean ± *SEM* *n* = 6 per group. *p < 0.05 (Student's *t* test).

Recent studies have revealed neurotrophic relationships among different striatal neurons. Modulatory striatal interneurons are engaged in reciprocal neurotrophic relationships with DA SNpc (Figure 1a). Adult FS and some striatal ACh produce glial cell line‐derived neurotrophic factor (GDNF; Hidalgo‐Figueroa, Bonilla, Gutiérrez, Pascual, & López‐Barneo, 2012), a potent dopaminotrophic factor required for survival and functionality of DA SNpc (Ibáñez & Andressoo, 2016; Kumar et al., 2015; Pascual et al., 2008). However, the role of GDNF in adult SNpc survival is still controversial (Kopra et al., 2015; Pascual & López‐Barneo, 2015) and needs further clarification. Concomitantly, Sonic hedgehog (Shh), an extracellular ligand involved in cellular specification during development (Ingham & McMahon, 2001), is produced by DA SNpc and is required during late embryogenesis and early postnatal life for the survival and phenotypic maintenance of FS and ACh as well as DA SNpc itself in a non‐cell‐autonomous manner (Gonzalez‐Reyes et al., 2012). However, no defect on striatal interneurons has been associated with the strong DA SNpc degeneration observed in Parkinson's disease, suggesting that further complexity is involved in postnatal neurotrophic maintenance of the striatal modulatory circuit. Thus, Shh chronic loss during development and after maturation of striatal neuronal populations may imply different outcomes.

To elucidate postnatal neurotrophic relations in the striatal modulatory circuit, we first investigated the involvement of DA SNpc in striatal interneuron survival by decreasing the number of DA SNpc during postnatal life using Cre‐loxP technology. Next, we evaluated the postnatal role of Shh in interneuron maintenance by genetically reducing the levels of Smoothened (Smo), a protein strictly required for transducing Shh signaling, in striatal ACh and FS and characterized the physiology and integrity of the striatal modulatory circuit. Finally, we searched for alternatives sources of Shh that could be involved in ACh postnatal maintenance.

## 2 RESULTS

### 2.1 Postnatal dependency of striatal FS but not ACh on DA SNpc

Previous studies suggested that Shh produced by DA SNpc is required for the maintenance of both striatal ACh and FS (Gonzalez‐Reyes et al., 2012). However, no alterations in the number of striatal interneurons have been associated with the progressive neurodegeneration of DA SNpc observed in Parkinson's disease (Fahn, 2009; Lang & Lozano, 1998). To evaluate the postnatal neurotrophic potential of DA SNpc over striatal interneurons, we decreased the number of dopaminergic neurons and, consequently, the production of Shh and other neurotrophic molecules synthesized by these cells (Figure 1a,b). We employed a previously published mouse model where the mitochondrial *Sdhd* gene is specifically inactivated in the DA SNpc (Diaz‐Castro et al., 2012). Th‐Sdhd mice showed a normal number of DA SNpc at birth but suffered a specific dopaminergic postnatal neurodegeneration as they acquired functionality. Six‐month‐old mice lacked more than 95% of DA SNpc, a reduction that remained stable in older mice and correlated with motor‐related behavioral defects and with a strong decrease in striatal DA levels (Diaz‐Castro et al., 2012). The authors also showed that the Th‐Sdhd model did not present any nonspecific recombination in striatal neurons and that MSN and FS were not altered at the initial stages of the neurodegeneration (2 months old; Diaz‐Castro et al., 2012). We extended this observation to older mice in order to establish the postnatal role of DA SNpc‐derived signals over the integrity of striatal interneurons. Stereological unbiased quantifications of ACh and FS in 8‐month‐old mice revealed a clear decrease (42%) in the number of striatal FS without altering the ACh population (Figure 1c). Although Th‐Sdhd mice presented reduced mobility and a difficulty for independent feeding, we were able to age a small group of mice observing a similar reduction in the number of FS interneurons in 12‐month‐old mice (21,772 ± 4,564 control vs. 13,276 ± 1870 Th‐Sdhd; *p* = 0.1; Student's *t* test; *n* = 3). These results point to the existence of a neurotrophic circuit involving the maintenance of FS by DA SNpc and the independence of the striatal ACh from the DA SNpc.

### 2.2 Survival of striatal ACh but not FS requires Shh signaling

**Figure 2:**
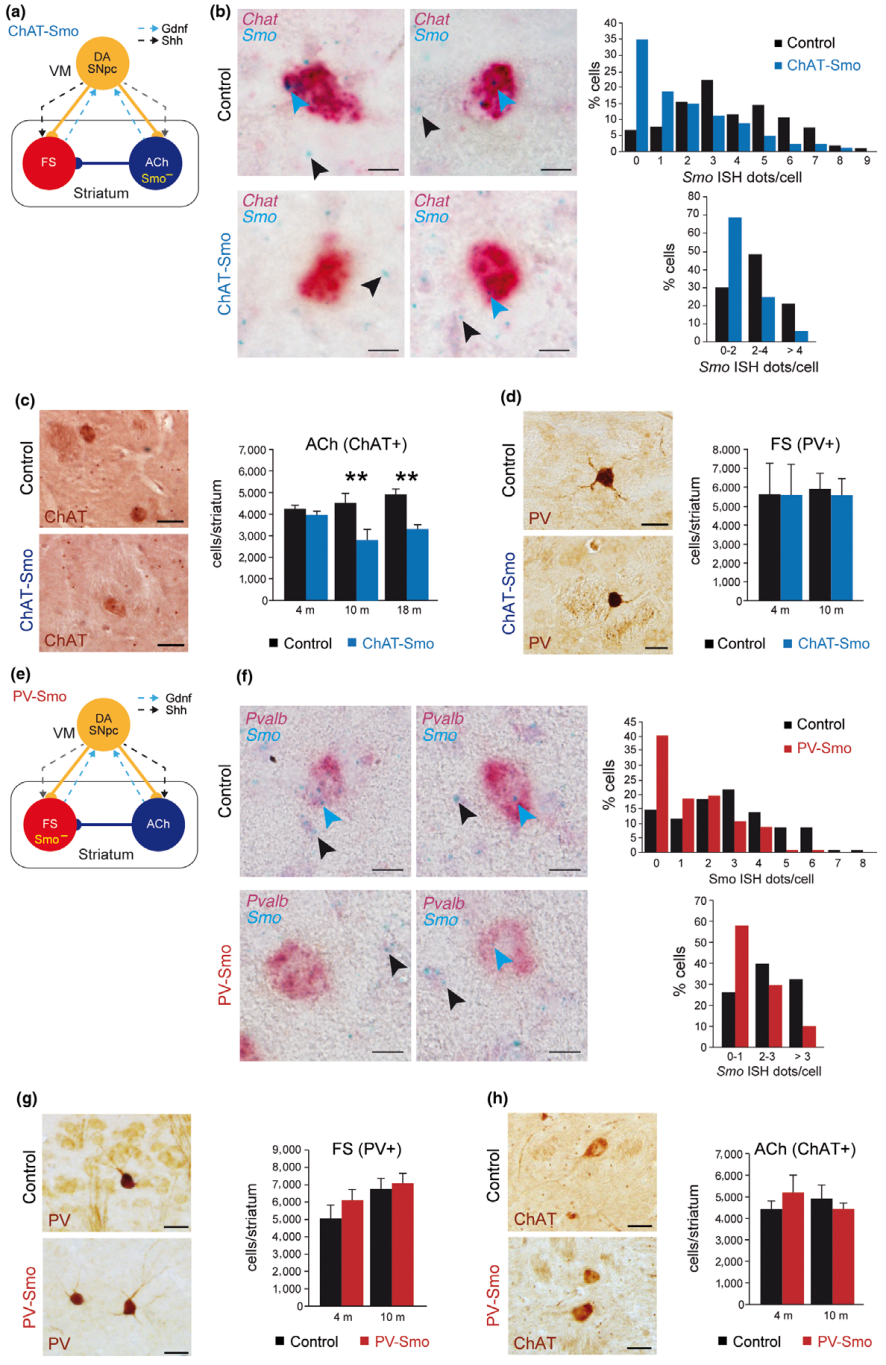
Shh is required for cholinergic (ACh), but not fast‐spiking (FS) striatal interneurons survival. (a, e) Schematic representation of the ChAT‐Smo (a) and PV‐Smo (e) mouse models, where Smo is specifically inactivated (Smo^−^) in ACh (a–d) or FS (e–h) interneurons. Solid lines represent neuronal connectivity and dashed lines neurotrophism. (b) In situ hybridization in coronal brain slices from 4‐month‐old (m) ChAT‐Smo mice using *Chat* (red) and *Smo* (blue) probes. Left panels show representative images. Blue arrowheads indicate *Smo* signal in *Chat*‐positive interneurons and black arrowheads depict *Smo* signal in non‐*Chat* cells. Scale bar: 10 µm. Right graphs represent the percentage of *Chat*‐positive interneurons with different *Smo* levels (individual values, up, or by ranges, down). 103 (control, black bars) and 80 (ChAT‐Smo, blue bars) cells from four independent mice. (c) Left: representative striatal coronal sections from control (top) and ChAT‐Smo (bottom) 10‐month‐old (m) mice immunostained with an anti‐ChAT antibody (a cholinergic interneuron marker). Scale bar: 50 µm. Right: number of ChAT‐positive interneurons per striatum of 4, 10, and 18 m control and ChAT‐Smo mice quantified by stereological methods. (d) Left: immunostaining anti‐PV in striatal coronal sections of control (top) and ChAT‐Smo (bottom) 10 m mice. Scale bar: 25 µm. Right: stereological quantification of the number of PV+ interneurons per striatum in control and ChAT‐Smo 4 and 10 m mice. In (c and d), *n* (4 m) = 3; *n* (10 m) = 4; and *n* (18 m) = 5 mice per group. (f) In situ hybridization in coronal brain slices from 4 m PV‐Smo mice using *Pvalb* (red) and *Smo* (blue) probes. Left panels show representative images. Blue arrowheads indicate *Smo* signal in *Pvalb‐*positive interneurons, and black arrowheads depict *Smo* signal in non‐*Pvalb* cells. Scale bar: 10 µm. Right graphs represent the percentage of *Pvalb*‐positive interneurons with different *Smo* levels (individual values, up, or by ranges, down). 115 (control, black bars) and 102 (PV‐Smo, red bars) cells from six independent mice. (g) Left: immunostaining anti‐PV in coronal striatal sections of 10 m control (up) and PV‐Smo (bottom) mice. Scale bar: 25 µm. Right: Number of total striatal PV+ cells from 4 and 10 m control and PV‐Smo mice. (h) Left: immunostaining anti‐ChAT in coronal striatal sections from control (up) and PV‐Smo (bottom) 10 m mice. Scale bar: 50 µm. Right: Number of total ChAT+ cells from control and PV‐Smo 4 and 10 m mice. In (g and h), *n* (4 m) = 5; *n* (10 m) = 4 mice per group. In the entire figure, values represent mean ± *SEM*. **p < 0.01 (two‐way ANOVA–Sidak's correction). VM: ventral mesencephalon

Removal of Shh produced by DA SNpc during development has a profound effect on the number and function of adult striatal ACh and FS (Gonzalez‐Reyes et al., 2012). To separately investigate the effect of a postnatal reduction in the Shh signaling on ACh or FS, we independently decreased Smo expression in each cell type. Shh binds to Patched homolog 1 or 2, which in turn relieves the repression of the serpentine transmembrane protein Smo, the obligatory Shh transducer (Ingham & McMahon, 2001). Striatal ACh and FS acquire their phenotype during the first 2–3 postnatal weeks in rodents along with the expression of specific markers such as choline acetyltransferase (ChAT, Ach; Phelps, Brady, & Vaughn, 1989) and parvalbumin (PV, FS; Schlösser, Klausa, Prime, & Bruggencate, 1999). To conditionally delete Smo in ACh or FS, we selected the ChAT‐Cre (Rossi et al., 2011) and PV‐Cre (Hippenmeyer et al., 2005) lines (Figure 2a,e). To validate the specificity of these mouse lines, we used the Rosa26R‐YFP reporter mouse. Almost all YFP‐positive cells coexpressed the ChAT marker by immunohistochemistry in ChAT‐Cre; R26R‐YFP mice (Supporting Information Figure S1a) and more than 50% of cells positive for PV were also positive for YFP in the PV‐Cre; R26R‐YFP line (Supporting Information Figure S1b), indicating specific recombination in both mouse models. To measure the efficiency of the Cre recombinase, we compared the striatal mRNA levels of *Smo* between control and *Chat^Cre/+^; Smo^loxP/loxP^* (ChAT‐Smo) mice or between control and *Pv^Cre/+^; Smo^loxP/loxP^* (PV‐Smo) mice. In the ChAT‐Smo model, we observed a trend to decrease in the levels of *Smo* mRNA at 1 month, and this was further confirmed at 4 and 10 months of age (Supporting Information Figure S1c). In the PV‐Smo model, we did not observe any changes in the mRNA levels of *Smo* at 1 month of age (Supporting Information Figure S1d). However, as time advanced, we found a significant decrease of around 50% in the striatal *Smo* levels (4‐month‐old mice), and this reduction was further confirmed at 10 months of age (Supporting Information Figure S1d). Notably, Smo is not only expressed within the striatum by ACh and FS but also by other neuronal and non‐neuronal cells (Gonzalez‐Reyes et al., 2012) that do not undergo recombination in our model, strongly suggesting that the reduction in *Smo* mRNA levels observed in the interneurons of both ChAT‐Smo and PV‐Smo mice was likely underestimated. To study the deletion of *Smo* in both Cre lines at single cell level, we performed double in situ hybridization (ISH) combining *Chat* (Figure 2b) or *Pvalb* (Figure 2f) with *Smo* probes. Quantification of ISH revealed that 70% of ACh cells in the ChAT‐Smo model and around 60% of FS interneurons in the PV‐Smo almost lost *Smo* expression and a big percentage of the other ACh or FS decreased Smo expression (Figure 2b,f), strongly indicating that our models produce enough recombination in the targeted cells to evaluate the function of Shh signaling. To determine whether the postnatal disruption of Shh signaling in either ACh or FS produced striatal neurodegeneration, we estimated the number of ChAT‐ (ACh) and PV‐ (FS) immunoreactive cells in the striatum using unbiased stereological methods. At 10 and 18 months of age, we observed a significant and consistent decrease (40% and 36%) in the number of ACh in the striatum of ChAT‐Smo mice (Figure 2c). This neurodegenerative process did not affect the survival of FS (Figure 2d). Interestingly, in the PV‐Smo model, the number of PV or ACh expressing cells in the striatum did not change at any of the ages analyzed when compared with control mice (4 and 10 months old; Figure 2g,h). To confirm that the PV‐Cre line can drive neurodegeneration in FS cells, we generated a *Pv^Cre/+^; Sdhd^loxP/−^* (PV‐Sdhd) model (Supporting Information Figure S2a). These mice developed a strong phenotype with a very reduced lifespan and body weight (Supporting Information Figure S2b,c) and fast neurodegeneration was observed in striatal FS (Supporting Information Figure S2d; 45 days old). As a control, we verified that the recombination in this new mouse model using the *Rosa26R‐Tomato* reporter was similar to control mice and did not produce any nonspecific recombination (Supporting Information Figure S1e).

**Figure 3:**
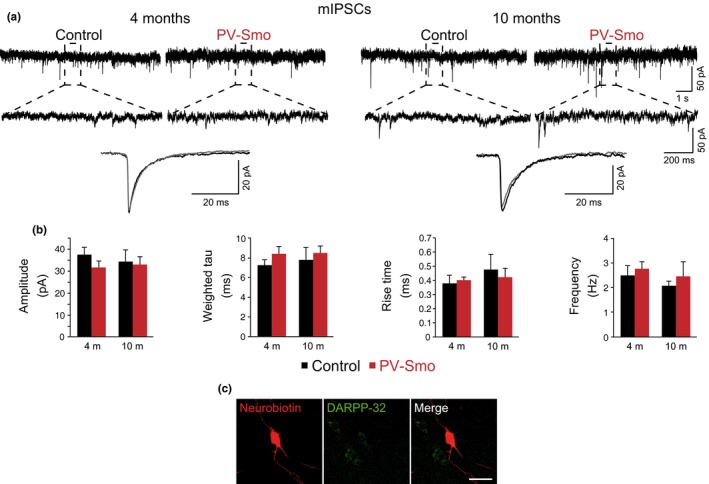
Postnatal ablation of Shh signaling in fast‐spiking interneurons does not alter GABAergic miniature inhibitory postsynaptic currents (mIPSCs) in striatal medium spiny neurons (MSN). (a) Traces illustrating whole‐cell recordings of GABAergic mIPSCs from MSN from 4 (*n* = 3; left panels)‐ and 10 (*n* = 3; right panels)‐month‐old control and PV‐Smo mice. Lower panels show traces illustrating mIPSCs averages from 4‐ and 10‐month‐old control (black traces) and PV‐Smo (gray traces) mice. (b) Quantification of mIPSCs parameters (amplitude, weighted tau, 20%–80% rise time and frequency) in control (black bars; 4 months old, *n* = 13 cells; 10 months old, *n* = 8 cells) and PV‐Smo (red bars; 4 months old, *n* = 15 cells; 10 months old, *n* = 9 cells) mice. (c) Photomicrograph showing a neurobiotin‐injected neuron and its colocalization with DARPP‐32 (dashed line). Scale bar represents 20 µm. In the entire figure, values represent mean ± *SEM* Student's *t* test

Although Shh was not needed for the survival of postnatal FS, we wondered if the decrease in Shh signaling could lead to any functional alteration in the striatum of PV‐Smo mice. We recorded GABAergic miniature inhibitory postsynaptic currents (mIPSCs) from striatal MSN, which receive a strong perisomatic inhibition from FS interneurons (Gittis et al., 2010). The amplitude, kinetic characteristics, and frequency of these mIPSCs were unaltered in 4‐ and 10‐month‐old PV‐Smo mice (Figure 3a,b), indicating that Smo deletion in FS does not have a strong effect over striatal function. Although MSN represent more than 95% of striatal neurons, we injected neurobiotin in a subset of the registered neurons to confirm their identity using a MSN marker, DARPP‐32 (Figure 3c).

Collectively, these data strongly suggest that Shh signaling is postnatally required in the striatum to preserve ACh but is not involved in the postnatal maintenance of FS.

### 2.3 Shh is expressed by striatal interneurons

**Figure 4:**
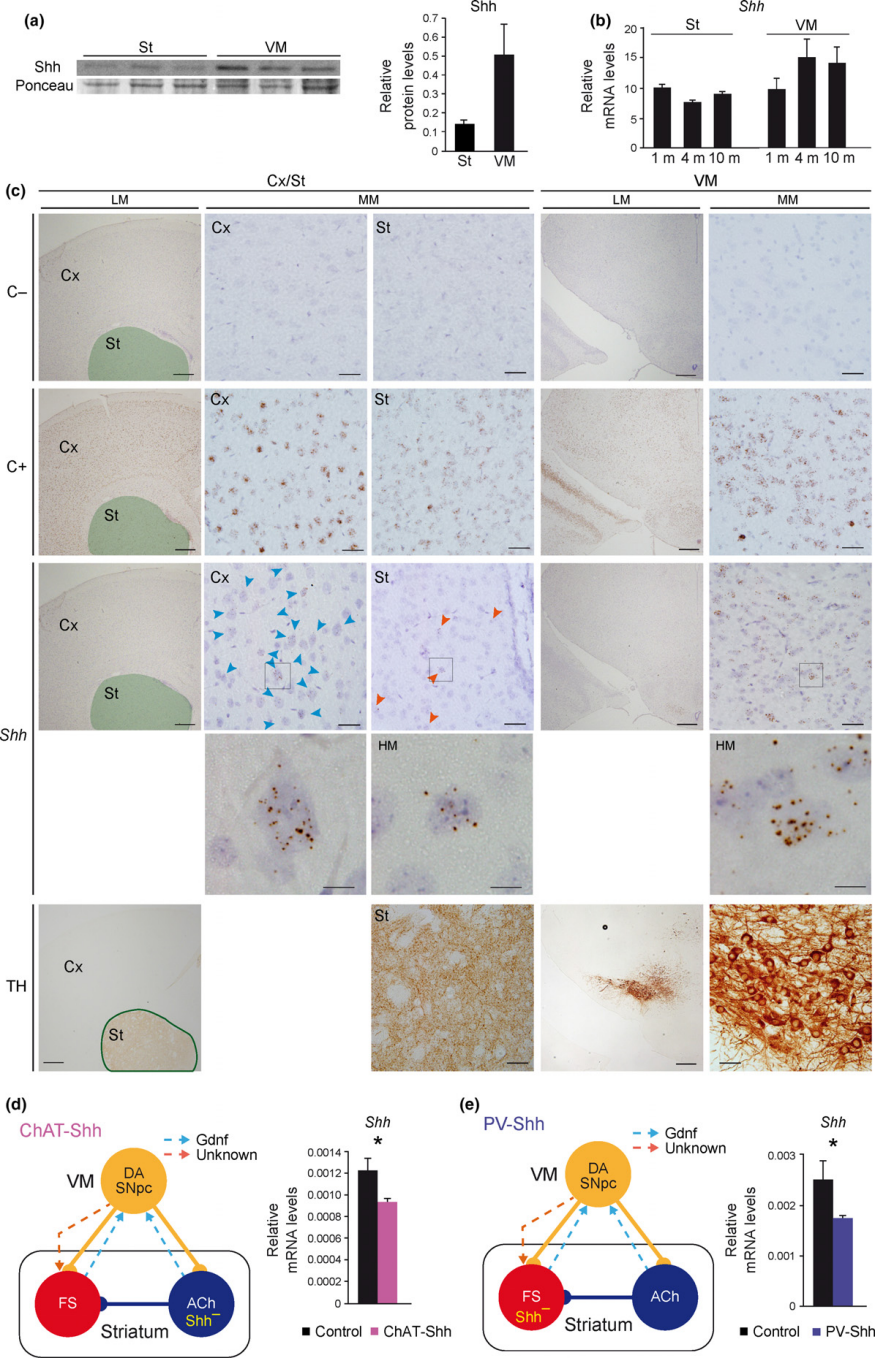
Cholinergic (ACh) and fast‐spiking (FS) interneurons contribute to striatal Shh production. (a) Shh protein levels by Western blot in striatum (St) and ventral mesencephalon (VM) from three wild‐type 2‐month‐old mice. Total levels of protein were measured by Ponceau staining. (b) *Shh* mRNA levels measured by qRT–PCR from St and VM of 1‐, 4‐, and 10‐month‐old (m) wild‐type mice. *n* (1 m) = 6; *n* (4 m) = 7 (St) and 5 (VM); and *n* (10 m) = 9 mice per group. (c) Representative coronal sections at cortical (Cx)‐striatal (St; green area in low magnification [LM] images) and VM levels from wild‐type 2‐month‐old mice in situ hybridized with a negative control probe (upper row; C−), a positive control probe (second row; *Ppib*; C+), and a *Shh* probe (third row). Low (LM), medium (MM), and high (HM; squares inside MM depicted below) magnification images are shown. In the fifth row, consecutive sections from the same brains have been immunostained with an anti‐TH antibody to identify the regions in situ hybridized in the upper rows. Scale bar: 400 µm in LM, 40 µm in MM, and 10 µm in HM images. (d, e) Left panels, schematic representation of the ChAT‐Shh (d) and PV‐Shh (e) mouse models, where Shh is specifically inactivated (Shh^–^) in ACh (d) or FS (e) interneurons. Right panels, *Shh* mRNA levels measured by qRT–PCR from the St of *Chat^Cre/+^; Shh^loxP/loxP^* (ChAT‐Shh; d) and *Pv^Cre/+^; Shh^loxP/loxP^* (PV‐Shh; e) mice. In (d and e), *n* (d) = 5 control and 4 mutant; *n* (e) = 6 control and 5 mutant mice per group. In the entire figure, values represent mean ± *SEM*. *p < 0.05 (Student's *t* test)

To reconcile the dependence of striatal ACh on Shh and not on DA SNpc for postnatal survival, we searched for alternative postnatal sources of Shh. Analysis of Shh protein levels by Western blot revealed a modest but detectable level in the striatum (Figure 4a). To differentiate between the Shh protein produced in the DA SNpc and transported to the striatum from those synthetized by local striatal cells, we measured *Shh* mRNA levels. Shh showed a similar expression level in the postnatal striatum and ventral mesencephalon of wild‐type mice (Figure 4b), suggesting that striatal cell types may be able to provide Shh to ACh during postnatal life in addition to the Shh supplied by DA SNpc. To confirm this result, we evaluated the expression of *Shh* mRNA in the postnatal striatum using in situ hybridization. Analysis of 3‐month‐old wild‐type mice revealed a prominent but widely distributed expression of *Shh* in cortical and SN brain areas and a highly restricted expression of *Shh* in the striatum (Figure 4c). *Shh* signal was limited to cells with large nuclei and was evenly distributed in the striatum (Figure 4c), two characteristics that are shared by both ACh and FS (Tepper & Bolam, 2004). To confirm the contribution of these interneurons to the striatal production of Shh, we conditionally deleted *Shh* in ACh or FS (*Chat^Cre/+^; Shh^loxP/loxP^* –ChAT‐Shh– and *Pv^Cre/+^*;* Shh^loxP/loxP^* –PV‐Shh–) and showed a significant reduction in the *Shh* mRNA levels in the striatum in both mouse models (Figure 4d,e) using qRT–PCR. Altogether, these results strongly suggest that both interneurons contribute to the local expression of Shh in the striatum.

### 2.4 ACh are required for postnatal DA SNpc maintenance in a GDNF‐independent manner

**Figure 5:**
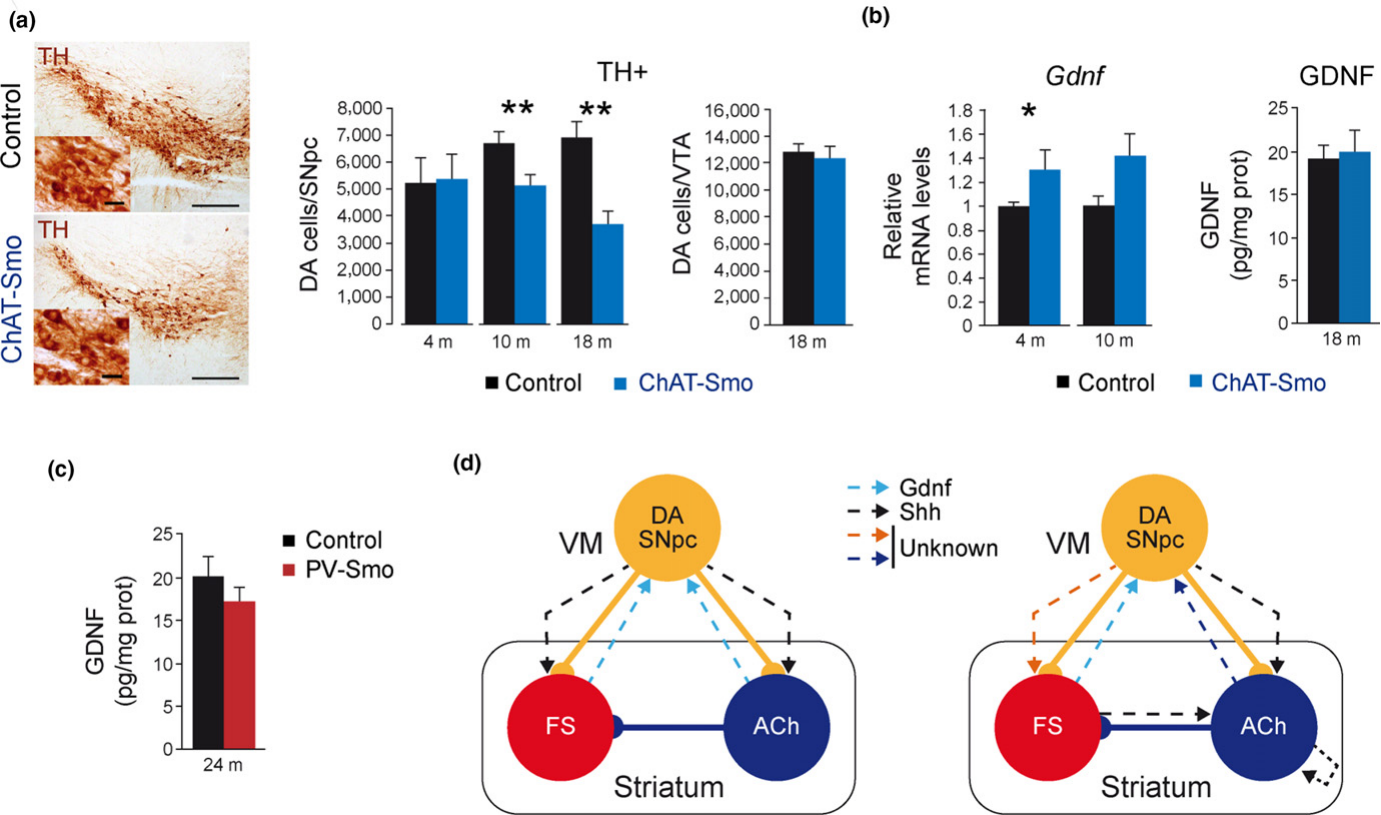
Neurodegeneration of DA SNpc after Shh signaling ablation in cholinergic interneurons. (a) Left: mesencephalic coronal sections immunostained with anti‐TH antibody (a DA SNpc marker) from 10‐month‐old (m) Control (left) and ChAT‐Smo (right) mice. Scale bars: 200 and 25 µm in the inset. Right: number of TH+ neurons in the SNpc of control and ChAT‐Smo mice measured by stereological methods. *n* (4 m) = 3; *n* (10 m) = 4; and *n* (18 m) = 5 mice per group. (b) GDNF mRNA (left graph) and protein (right graph) levels measured by qRT–PCR and ELISA in total striatum from control and ChAT‐Smo 4, 10, and 18 m mice. Data were normalized to housekeeping gene *Actb* expression in RNA and total protein content in ELISA. RNA: *n* (4 m) = 4 and *n* (10 m) = 5 mice per group. ELISA: *n = *4 mice per group. (c) GDNF protein levels measured by qRT–PCR and ELISA in total striatum from control and PV‐Smo 24 m mice. Data were normalized to total protein content. ELISA: *n = *6 (control) and 4 (PV‐Smo) mice. In the entire figure, values represent mean ± *SEM*. *p < 0.05; **p < 0.01 (ANOVA‐LSD in (a) or Student's *t* test in (b, c)). (d) Left scheme represents the previous symmetric model of the postnatal striatal modulatory circuit neurotrophism and the right scheme depicts the working model based on the present work. Solid lines represent neuronal connectivity and dashed lines neurotrophism. VM: ventral mesencephalon

Simultaneous degeneration of striatal ACh and FS is correlated with the death of the DA SNpc, which is attributed to a decrease in striatal GDNF production (Gonzalez‐Reyes et al., 2012). To estimate whether a postnatal reduction in the number of striatal ACh was correlated with the neurodegeneration of the DA SNpc in the absence of FS neurodegeneration, we estimated the number of tyrosine hydroxylase (TH) immunoreactive DA SNpc in ChAT‐Smo mice (Figure 2a). We detected a reduction of about 20% in 10‐month‐old mice but not at earlier time points (Figure 5a), which was also confirmed and increased in 18‐month‐old mice (36%; Figure 5a). Interestingly, no changes were observed in the DA ventral tegmental area (VTA) neurons in the ChAT‐Smo mice (Figure 5a). Although the decrease in the number of DA SNpc was modest to produce neurochemical alterations, we estimated the DA levels in the striatum using HPLC. No changes were observed in the ChAT‐Smo or the PV‐Smo models (Supporting Information Figure S3a,b), something that could be expected as the dopaminergic system compensate fast the loss of DA SNpc by increasing the local production of the DA by the remaining cells (Golden et al., 2013; Zigmond, Abercrombie, Berger, Grace, & Stricker, 1990; Zigmond, Acheson, Stachowiak, & Strickerm, 1984).

GDNF may be involved in postnatal DA SNpc maintenance (Kopra et al., 2015; Pascual & López‐Barneo, 2015; Pascual et al., 2008). Although FS are the main producers of striatal GDNF, ACh marginally contribute to its expression (Hidalgo‐Figueroa et al., 2012). To test whether GDNF contributes to the degeneration of DA SNpc observed in the absence of Shh signaling in ChAT‐Smo mice, we estimated striatal *Gdnf* mRNA by qRT–PCR. As shown in Figure 5b, the levels of *Gdnf* were not decreased but increased at 4 months of age and a trend to increase was also observed at 10 months of age, a compensatory phenomenon that has been observed in other models of DA SNpc injury (Hidalgo‐Figueroa et al., 2012). To further confirm that the neurodegeneration of the DA SNpc did not involve GDNF alteration, we estimated its protein levels in 18‐month‐old mice. ELISA measurements did not reveal any differences between control and ChAT‐Smo mice in the GDNF striatal levels (Figure 5b). As a technical control, we measure GDNF levels in PV‐Smo (a model without FS defects; Figure 2e) and PV‐Sdhd mice (Supporting Information Figure S2e) and observed no changes in the PV‐Smo (Figure 5c) and a clear decrease in the striatal levels of GDNF in the PV‐Sdhd model (Supporting Information Figure S2e), which correlated with a reduction in the number of FS in those mice (Supporting Information Figure S2d). All these data strongly suggest that Shh signaling is required to maintain ACh in the postnatal striatum and that these cells are necessary for the integrity of DA SNpc in a GDNF‐independent manner.

### 2.5 Shh does not regulate GDNF production

A negative feedback loop between striatal GDNF and DA SNpc Shh expression has been described (Gonzalez‐Reyes et al., 2012). To test whether the different genetic manipulations used in this work could produce any alteration in the ventral mesencephalic Shh levels, we estimated its mRNA levels using qRT–PCR. None of the genetic model presented changes in Shh levels (Supporting Information Figure S4a).

Striatal injections of Shh agonist cyclopamine (Cyc) and antagonist SAG produced a strong decrease (SAG) or increase (Cyc) in the striatal GDNF levels estimated by qRT–PCR (Gonzalez‐Reyes et al., 2012). In the light of the absence of regulation between GDNF and Shh observed in our models (Supporting Information Figure S4a), we tried to replicate the effect of SAG and Cyc over GDNF expression. No differences were found after stereological injection of both molecules using the protocol described in Ref. (Gonzalez‐Reyes et al., 2012), although a trend to decrease with SAG treatment was detected (Supporting Information Figure S4b), altogether suggesting that the cross‐regulation between GDNF and Shh may be of smaller magnitude than previously proposed.

## 3 DISCUSSION

We define three parallel and interdependent neurotrophic relationships characterized by (a) a bidirectional relation between DA SNpc and FS which may involve GDNF (Hidalgo‐Figueroa et al., 2012; Kopra et al., 2015; Pascual & López‐Barneo, 2015; Pascual et al., 2008); (b) a unidirectional relation between ACh and DA SNpc that does not involve GDNF; and (c) a dependence of ACh survival on Shh that not exclusively requires the DA SNpc but may imply FS and ACh (Figure 5d).

Our experiments demonstrate that DA SNpc innervation is required for FS but not ACh survival. These results are compatible with the observed changes in the brain of PD patients, where the cholinergic tone was not decreased but was instead increased in the striatum (Girasole & Nelson, 2015). Our results are also compatible with the effect provoked by acute DA SNpc intoxication with 6‐hydroxydopamine over the striatal modulatory circuit. In this model, the cholinergic innervation of MSNs is increased and the number of GABAergic feedforward inhibition projections from FS interneurons reaching MSNs is decreased (Salin et al., 2009). This initial loss of striatal GABAergic synapses after DA SNpc cell death stimulates a compensatory adaptation in the cholinergic projections. Our results suggest that the sustained reduction in the striatal dopamine levels could finally induce not only the degeneration of the GABAergic terminals but also the death of FS interneurons. Notably, Th‐Sdhd mice do not present alterations in the number of FS at an early age (2‐month‐old mice; Diaz‐Castro et al., 2012), suggesting either that the striatal degeneration proceeds slowly after DA SNpc death or that a strong decrease in the number of DA SNpc is required to alter the FS population. In agreement with mouse models, there are no reports of FS alteration in the striatum of PD patients, implying either that DA SNpc loss is not enough in the patients to provoke a decrease in the number of FS interneurons or that the dopamine replacement therapy used to treat those patients could prevent the loss of these cells. The first option is unlikely as DA SNpc loss is estimated to be 60%–70% at the onset of symptoms (Fearnley & Lees, 1991; Lang & Lozano, 1998). Favoring the second option is the report that, after binding to D_2_ receptors, dopamine can be internalized to form a signaling complex (including ß‐arrestin and protein phosphatase 2) that regulates the Akt pathway (Beaulieu et al., 2008), a cascade involved in neuroprotection (Dudek et al., 1997; Soler et al., 1999). Dopamine inhibits GABA_A_‐mediated synaptic inputs to intrinsic striatal neurons (Bracci, Centonze, Bernardi, & Calabresi, 2002; Momiyama & Koga, 2001; Pisani, Bonsi, Centonze, Calabresi, & Bernardi, 2000) through presynaptic D_2_ receptors (Centonze et al., 2003; for a review, see Berke, 2011). All together, we speculate that dopamine could be involved in the acute and chronic protection of FS striatal terminals and, therefore, could contribute to long‐term FS survival. Although the Th‐Sdhd model shows postnatal neurodegeneration of the DA SNpc (Diaz‐Castro et al., 2012), we cannot exclude subtle developmental alterations that could produce postnatal phenotypes. Posterior studies with this and other models of DA SNpc postnatal depletion will be required to validate our results and provide mechanistic insights. However, no tamoxifen‐regulated Cre line has so far proven efficiency in recombining postnatal DA SNpc, something that could be overcome with bitransgenic systems containing Th or Pitx3‐tet, and TRE‐Cre lines (Chinta et al., 2007; Lin et al., 2012; Tillack, Aboutalebi, & Kramer, 2015).

Shh signaling has been proposed as a key factor for the physiology and survival of each component of the striatal modulatory circuit. In this context, the work by Gonzalez‐Reyes et al. (2012) showed that the embryonic removal of Shh from DA SNpc produces degeneration of striatal ACh and FS (Figure 5d, left). However, our data reveal that postnatal interruption of Shh signaling in FS cells has no effect on interneuron survival or striatal physiology and that, on the contrary, Shh signaling is required in striatal ACh for survival (Figure 5d, right). The fact that the recombination achieved in the PV‐Smo models is moderated (around 60% cells loss Smo expression) could indicate the presence of two independent FS interneurons in the striatum with different levels of PV expression, something that was recently shown in single cortical cell RNA‐seq studies (Zeisel et al., 2015). Therefore, it could be possible that a subpopulation of the FS could be dependent on Shh signaling. In agreement with that possibility, we observed a similar decrease in the number of FS in the Th‐Sdhd model at different time points (40%), suggesting that only a subpopulation of FS could depend on DA SNpc. The apparent contradiction between our data and the one generated by Gonzalez‐Reyes et al (2012) can be reconciled by a dual role of Shh, as a neuronal specification factor during development for both FS and ACh, and as a survival signal for ACh during postnatal life. Interestingly, Shh is required during development for the specification of several neuronal populations, including DA SNpc. Shh is embryonically secreted from the ventral floor and basal plates, and the absence of Shh leads to a reduction in the number of DA SNpc (Blaess, Corrales, & Joyner, 2006). The described expression of Shh in DA SNpc (Gonzalez‐Reyes et al., 2012) combined with our results suggest a plausible role for Shh in the specification of striatal interneurons. In the absence of Shh during development, striatal interneurons are disturbed (Gonzalez‐Reyes et al., 2012); however, after this critical developmental period, our results demonstrate that the absence of Shh signaling is less dramatic to postnatal striatal interneurons. Similarly, Shh signaling is not required for the survival of DA SNpc in a cell‐autonomous manner after E16 in mice (Zhou et al., 2016), but it is required at this late developmental time for functional specification of DA SNpc (Zhou et al., 2016). Interestingly, Shh has a distinct role over other cholinergic neurons, as an enhancer of postmitotic survival of basal forebrain cholinergic neurons (Reilly, Karavanova, Williams, Mahanthappa, & Allendoerfer, 2002) and as differentiation factor for motor neurons (Ericson, Morton, Kawakami, Roelink, & Jessell, 1996).

The endurance of postnatal ACh to DA SNpc loss but not to the absence of the Shh signaling pathway suggests the existence of additional Shh sources that guarantee the survival of these crucial interneurons. Our experiments demonstrate local Shh production in the striatum, partially driven by FS and ACh, that may add to the Shh produced by DA SNpc to maintain striatal ACh. In the context of PD, our model also predicts that ACh will survive in the absence of DA SNpc, but that inhibition of Shh signaling could accelerate neuronal degeneration. Therefore, a proposed PD therapy of pharmacological inhibition of the Shh pathway (Gonzalez‐Reyes et al., 2012), should be re‐evaluated.

Finally, we have described the dependence of DA SNpc on ACh in a process that, interestingly, does not require GDNF because neurodegeneration of these cells is observed in the absence of any striatal decrease in GDNF levels. Those results are in agreement with our previous observation that FS are the main striatal GDNF producers with only a marginal contribution by ACh (Hidalgo‐Figueroa et al., 2012).

Overall, we describe a new working model for maintenance of the postnatal striatal modulatory circuit that paves the way for researching new protective pathways that could be relevant for the normal physiology of these brain nuclei and for the fight against neurodegenerative disorders.

## 4 EXPERIMENTAL PROCEDURES

### 4.1 Transgenic mice

Transgenic mice were housed under temperature‐controlled conditions (22°C) in a 12‐hr light/dark cycle with access ad libitum to food and water. All experiments were performed according to institutional guidelines approved by the ethics committee of the Hospital Universitario Virgen del Rocio and the European Community (Council Directive 86/609/EEC). *Pv^Cre^* (Hippenmeyer et al., 2005), *Chat^Cre^* (Rossi et al., 2011), *Smo^Flox^* (Long, Zhang, Karp, Yang, & McMahon, 2001), *Shh^Flox^* (Lewis et al., 2001), *Rosa26R‐Tomato* (Madisen et al., 2010), and *Rosa26R‐YFP* mice (Srinivas et al., 2001) were obtained from Jackson Laboratory (Stocks numbers 8,069, 6,410, 4,526, 4,293, 7,909, and 6,148) and were maintained on their genetic background (B6;129P2, C57BL/6, 129X1/SvJ, and C57BL/6J). Breeding previous lines, we generated *Pv^Cre/+^; Smo^loxP/loxP^* (PV‐Smo), *Chat^Cre/+^*; *Smo^loxP/loxP^* (ChAT‐Smo), *Pv^Cre/+^*; *Shh^loxP/loxP^* (PV‐Shh), and *Chat^Cre/+^*; *Shh^loxP/loxP^* (ChAT‐Shh). *Th^Cre/+^*; *Sdhd^loxP/−^* (Th‐Sdhd) generation was described in Diaz‐Castro et al., 2012. *Rosa26R‐YFP* mice were crossed with *Pv^Cre/+^* and *Chat^Cre/+^* in order to identify cells that have undergone recombination. *Pv^Cre/+^*; *Sdhd^–/loxP^* (PV‐Sdhd) mice were generated from the original *Sdhd* KO (Piruat, Pintado, Ortega‐Saenz, Roche, & Lopez‐Barneo, 2004) and (Diaz‐Castro et al., 2012) floxed alleles. All the transgenic alleles were genotyped following Jackson Laboratory instructions. In Th‐Sdhd and PV‐Sdhd models, controls were always heterozygous for Sdhd (*Sdhd^−/flox^* without Cre recombinase or *Sdhd^−/+^* with Cre recombinase) to fairly compare with the conditional mice (*Cre*; *Sdhd^−/F^*).

### 4.2 Antibodies

The following primary antibodies were used in this study: antiparvalbumin (PV; rabbit, Swant, 1:1,000); anticholine acetyltransferase (ChAT; mouse, Millipore, 1:500); antityrosine hydroxylase (TH; rabbit, Novus, 1:1,000); chicken anti‐EGFP (chicken, Aves Lab, 1:1,000); rabbit anti‐EGFP (rabbit, Invitrogen, 1:1,000); and anti‐mCherry (Chicken, EnCor, 1:1,000).

### 4.3 Tissue preparation

After deep anesthesia with thiobarbital (Braun), mice were transcardially perfused with 0.1 M phosphate‐buffered saline (PBS), pH 7.4, followed by 4% paraformaldehyde (PFA; Sigma) in 0.1 M PBS, pH 7.4. Brains were immediately removed, postfixed overnight in the same fixative at 4ºC, and paraffin‐embedded using an automatic tissue processor (ASP300S; Leica). Coronal mouse brain sections of 20 µm were obtained using a microtome (RM2255; Leica) and were then incubated overnight at 37°C before immunohistochemistry.

For monitoring Cre recombinase expression, brains were embedded in gelatin and 50‐µm‐thick sections were obtained using a vibratome (Leica).

For in situ hybridization, 2‐month‐old wild‐type mice were processed following RNAscope protocols (ACD). In brief, immediately following dissection the brain was fixed in 10% neutral‐buffered formalin (Sigma) for 24 hr at room temperature. Fixed samples were paraffin‐embedded using an automatic tissue processor (ASP300S; Leica).

### 4.4 Light microscopy immunohistochemistry

Serial sections from control and transgenic mice were processed in parallel for light microscopy immunostaining using the same batches of solutions to minimize variability in the immunohistochemical labeling conditions. Coronal brain sections were deparaffinized using standard procedures, and the rehydrated sections were first treated with 3% H_2_O_2_ in PBS, pH 7.4 for 20 min to inhibit endogenous peroxidases, followed by 10% normal goat serum and bovine serum albumin (10 mg/ml) for 1 hr, to block unspecific binding sites. Sections were immunoreacted with one or two of the primary antibodies over 24 hr at 4°C. The tissue‐bound primary antibody was then detected by incubating for 1 hr with the corresponding fluorescent secondary antibody (Invitrogen, 1:800) or with the Envision‐Flex kit secondary antibody (1:1,000; DAKO). For double nonfluorescent analysis, 3‐3‐diaminobenzidine tetrahydrochloride (DAB; DAKO) and ß‐amino‐9‐ethyl‐carbazole (AEC; DAKO) substrates were used.

Sections were then air‐dried, dehydrated in graded ethanol, cleared in xylene, and coverslipped with DPX (BDH) mounting medium. For double immunofluorescence labeling, sections were coverslipped in Fluoromount (Sigma) and air‐dried at room temperature. Imaging was performed with a BX‐61 microscope (Olympus) or with a A1R Confocal (Nikon).

### 4.5 In situ hybridization

Coronal mouse brain sections of 10 µm were obtained using a microtome (RM2255; Leica) and were then incubated overnight at 37°C before ISH. RNAscope protocol was performed as indicated for formalin‐fixed paraffin‐embedded samples (ACD) using a HybEZ oven (ACD) with a negative control probe, a *Ppib* probe as a positive control, and *Shh*, *Pvalb*, *Chat*, and *Smo* probes (ACD). Individual detection was performed using the RNAscope 2.5 Assay and double ISH using the RNAscope 2.5 Duplex detection kit. Quantification of *Smo* signal was performed blinded to the genotypes by scoring the number of small dots (one point) in *Chat‐* or *Pvalb*‐positive interneurons. Bigger dots were scored with 2–4 points depending on its size.

### 4.6 Stereological analysis

The total number of FS (PV‐immunoreactive) and ACh (ChAT‐immunoreactive) was obtained by stereology‐based quantification of the striatum from different transgenic mice (*n* is described in the figure legends) using an Olympus BX61 microscope and the newcast software package (Olympus). The striatum was divided into dorsal and ventral areas and 25% of the total dorsal area was randomly covered by 26,738.7 µm^2^ dissectors per hemisphere. The total number of TH‐immunoreactive DA SNpc and VTA was similarly obtained in 20‐µm coronal microtome sections. About 20% of the total area was covered by 7,130.3 µm^2^ dissectors per hemisphere. Each analysis was carried out by a single examiner blinded to sample identities.

### 4.7 Quantitative RT–PCR

To determine mRNA levels, brain RNA was extracted from the striatum or ventral mesencephalon using TRIzol reagent (Invitrogen) in a homogenizer system (Bullet Blender; Next Advance Inc.) at speed 8, 3 min, 4°C. Five hundred nanogram of RNA was copied to cDNA using the qScript enzyme (Quanta Biosciences) in a final volume of 20 µl. Real‐time PCR was performed in Viia‐7 System (Applied Biosystems) using iTaq Universal Probes Supermix (*Gdnf, Smo*; Bio‐Rad). We used thermocycler conditions recommended by the manufacturer. PCRs were conducted in duplicates in a total volume of 20 µl containing 0.5 µl of the reverse transcription reaction. In each sample, *Gapdh* and *Actb* RNA levels were estimated to normalize for RNA input amounts. To normalize mRNA levels in knockout mice to those in control samples within the same age, we calculated an average cycle threshold of the control samples‐age group and processed all the samples in the age group to this average cycle threshold. Primers and probes are available upon request.

### 4.8 HPLC

Striata were dissected in ice‐cold PBS. After sonication, homogenates were filtered by Ultrafree‐MC centrifuge filter units (Millipore) and kept at −80ºC. Dopamine and related metabolites were analyzed by high‐performance liquid chromatography (HPLC) using a chromatographic ALB‐215 column (ANTEC Leyden) according to the manufacturer's indications. Protein pellets were resuspended in NaOH 0.1 N and quantified using Bradford reagent (Quick Start Bradford, Bio‐Rad).

### 4.9 GDNF ELISA

Striatal GDNF protein content was estimated using a commercial ELISA kit (GDNF Emax Immunoassay System; Promega). Brain was removed and immediately frozen in liquid nitrogen. Hemicortex and striatum were processed as described (Pascual et al., 2008). Absorbance from hemicortical sample extracts was subtracted to each individual striatal measurement.

### 4.10 Preparation of acute brain slices

For the preparation of brain slices, we used the N‐methyl‐d‐glucamine (NMDG) protective recovery method described by the laboratory of Guoping Feng (Peça et al., 2011; Ting, Daigle, Chen, & Feng, 2014; Zhao et al., 2011). In brief, 4‐ and 10‐month‐old mice of both sexes were anesthetized with 2% tribromoethanol (0.15 ml/10 mg) and rapidly decapitated. The brains were dissected and transferred to NMDG ice‐cold artificial cerebrospinal fluid (ACSF) composed of (in mM): 93 NMDG, 2.5 KCl, 1.2 NaH_2_PO_4_, 30 NaHCO_3_, 25 d‐glucose, 20 HEPES, 5 Na‐ascorbate, 2 thiourea, 3 Na‐pyruvate, 10 MgSO_4_, and 0.5 CaCl_2_. The pH of the solution was titrated to pH 7.3–7.4 with concentrated HCl (osmolality 310–315 mosmol/kg) and bubbled with carbogen (5% CO_2_–95% O_2_). Three hundred and fifty micrometer of coronal slices was cut on a Vibratome VT1200S (Leica) and transferred for initial recovery to NMDG ACSF at 33 ± 1°C for 10–15 min. Finally, slices were placed in a holding chamber at room temperature with normal ACSF composed of (in mM): 126 NaCl, 2.5 KCl, 2 CaCl_2_, 2 MgCl_2_, 1.25 NaH_2_PO_4_, 26 NaHCO_3_, and 10 d‐glucose (osmolality 305–315 mosmol/kg), pH 7.4, when bubbled with carbogen (5% CO_2_–95% O_2_).

### 4.11 Electrophysiology

For whole‐cell patch‐clamp recordings, slices were transferred into a recording chamber that was perfused with 33 ± 1°C bubbled ACSF at 2–3 ml/min. Putative striatal MSNs were visualized by a Nikon Eclipse FN1 microscope, a 40× water immersion objective (Nikon), and a USB 2.0 monochrome camera (DMK 31BU03.H, TheImagingSource). Whole‐cell recordings were performed using a double patch‐clamp EPC10 plus amplifier (HEKA). Under voltage‐clamp conditions, the patch pipettes for inhibitory postsynaptic currents (IPSCs) recording contained (in mM): 140 CsCl, 2 MgCl_2_, 0.05 EGTA, and 10 HEPES, adjusted to pH 7.2 with CsOH (280–290 mosmol/kg). Recording of miniature IPSCs (mIPSCs) was carried out in the presence of tetrodotoxin (1 μM) and kynurenic acid (2 mM) to block sodium channels and ionotropic glutamate receptors, respectively. Cells were held in voltage‐clamp mode at a holding potential (V_hold_) of −70 mV, while resistance was compensated by 70% (lag 10 μs). Recordings were discontinued if series resistances increased by >50% or exceeded 15 MΩ. Currents were low‐pass‐filtered at 3 kHz, digitized at 20 kHz, and acquired using PatchMaster software (HEKA). All miniature postsynaptic currents were analyzed with the program Stimfit (Guzman, Schlögl, & Schmidt‐Hieber, 2014). Recordings were first digitally filtered at 1 kHz. For each cell, all events were inspected to avoid false positives, and then, an average of all events detected was taken.

### 4.12 Identification of neurobiotin‐injected neurons

A subset of experiments was performed by including 0.5% neurobiotin (Vertor Laboratories) in the internal pipette solution. These slices were processed for revealing neurobiotin and its colocalization with DARPP‐32, a marker for MSN. After recording, slices (350 µm) were transferred to a 4% PFA in PBS solution at 4ºC overnight. Then, slices were washed with PBS and incubated with a blocking solution containing 3% fetal bovine serum and 1% Triton X‐100 in PBS for 2–3 hr. Then, slices were incubated with primary polyclonal antibody rabbit anti‐DARP‐32 (1:1,000; Millipore) and rhodamine avidin D (2 µl/ml; Vector Laboratories) overnight at 4°C. After washing with PBS, slices were incubated with secondary antibody (Alexa 488 donkey anti‐rabbit, 1:500, Jackson Immunoresearch) in PBS and 0.3% Triton X‐100 at 4°C overnight. Finally, slices were embedded with fluorescent mounting medium (Dako) and visualized in a confocal microscope (Zeiss LSM 7 Duo).

### 4.13 Western blot

The striatum and ventral mesencephalon from one hemisphere were dissected on PBS, pH 7.4 on ice, and fast‐frozen on liquid nitrogen. Samples were homogenized in RIPA buffer (NP‐40 1%; deoxycholate acid sodium salt 0.5%; SDS 0.1%; NaCl 150 mM; Tris–HCl 50 mM pH 7.4; EDTA 1 mM; protease and phosphatase inhibitors) using a 23‐G needle. Samples were centrifuged for 15 min at 13,000 g, 4°C, and pellet was resuspended in 100 µl of Laemmli buffer (Tris–HCl 250 mM pH 6.8; SDS 4%; glycerol 25%; bromophenol blue 0.1%; b‐mercaptoethanol 0.05%). The samples were sonicated and quantified using RCDC protocol (following manufacturer indications). The antibody used was anti‐Shh (5E1 DSHB), and the total level of proteins loaded was calculated by Ponceau staining. Quantification was carried out using IQ TL Software (v2003).

### 4.14 Stereotactic injection

Mice were anaesthetized using a solution of ketamine/xylazine at 100, 8 mg/kg, respectively. SAG and CYP were unilaterally injected into the right striatum (−0.5 mm anteroposterior; ±2.4 mm mediolateral; −2.5 mm dorsoventral) using a 1‐µl neurosyringe (7001 KH; Hamilton). The needle was slowly lowered to coordinates, and it was left in place for 5 min after the injections before it was slowly withdrawn. Cyclopamine (C‐8700; LC Laboratories) was diluted at 2 µg/µl in 45% HBC (2‐hydroxypropyl‐beta‐cyclodextrin; Sigma) in PBS, and 0.5 µl of the prior solution was injected. Smoothened Agonist SAG (Calbiochem) was diluted in PBS at 50 nM and 32 nl of the prior solution was injected. Left striatum was injected with Sham solution (vehicle). Animals were sacrificed 30 hr after injections, striata were dissected, and RNA was extracted.

### 4.15 Statistical analysis

Data are presented as mean ± standard error of the mean. In the case of one variable, statistical significance was assessed by Student's *t* test with a Levene test for homogeneity of variance (normal distribution) or by the nonparametric Mann–Whitney *U* test (non‐normal distribution). For two variables, data were analyzed by two‐way ANOVA followed by Sidak's multiple comparisons test. Survival curves were analyzed by Mantel–Cox test. graphpad prism 5.0 and spss 22.0 Software were used for statistical analysis.

## ETHICAL APPROVAL

Our animal research protocol was approved by the Animal Research Committee at our institution (“Establecimientos de cría, suministradores y usuarios de animales de experimentación de la Comunidad Autónoma de Andalucía” number ES41091008015 SE/15/CS/U). All procedures were conducted in agreement with the animal care guidelines of European Community Council (86/60/EEC).
