# Supplementary figures and images for: Substantia nigra dopaminergic neurons and striatal interneurons are engaged in three parallel but interdependent postnatal neurotrophic circuits
Source: Aging Cell. 2018 Jul 30;17(5):e12821. doi: 10.1111/acel.12821 (PMC6156350; doi:10.1111/acel.12821)

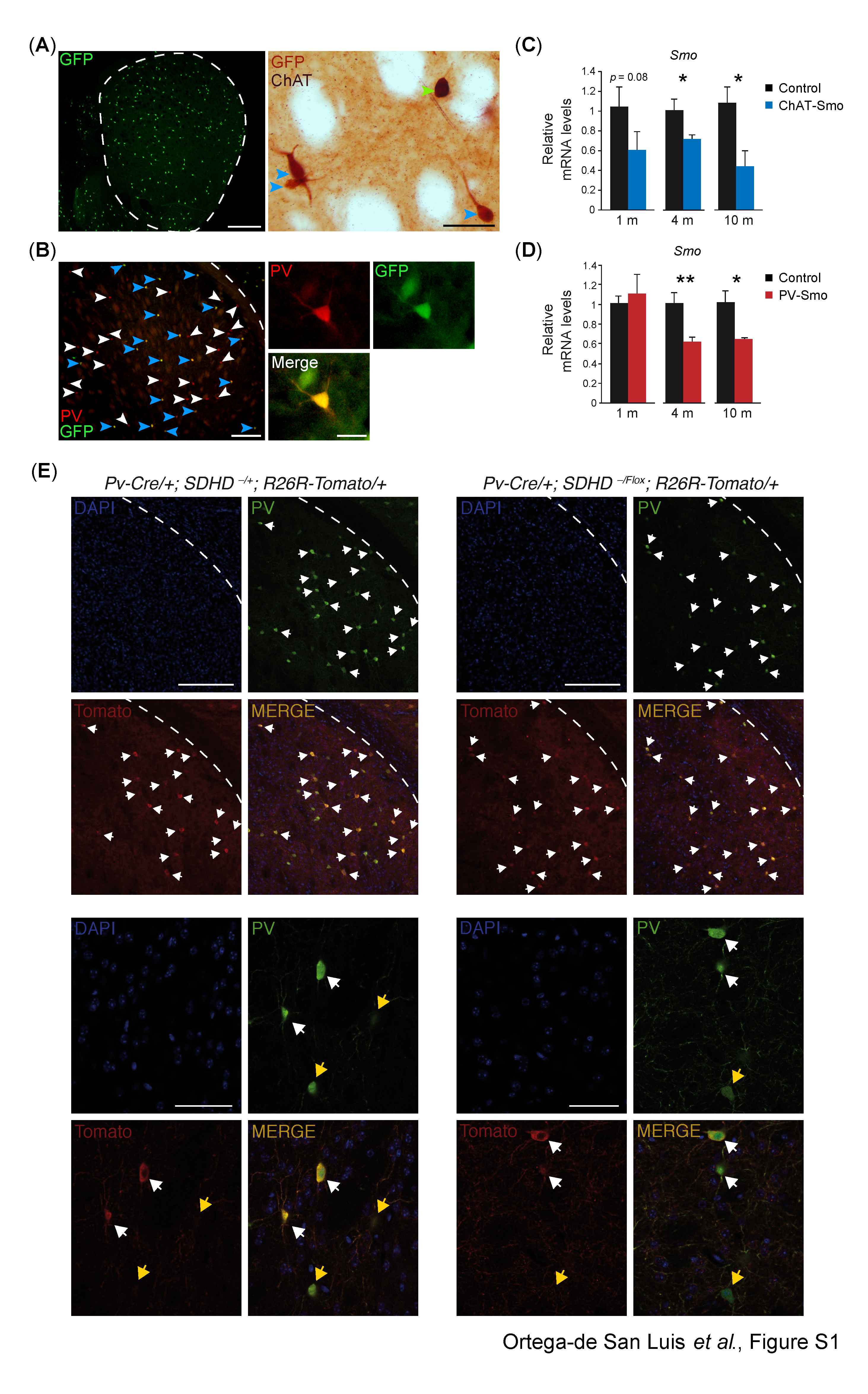

Supplement: Supplementary file 1 [file ACEL-17-e12821-s001.tiff]
